# Serum-MiR-CanPred: deep learning framework for pan-cancer classification and miRNA-targeted drug discovery

**DOI:** 10.1080/15476286.2025.2577433

**Published:** 2025-10-30

**Authors:** Naisarg Patel, Ankita Lawarde, Suhas Manikant Surisetti, Premkumar Thiruselvam, Prakash Lingasamy, Vino Sundararajan, Sajitha Lulu S, Andres Salumets, Vijayachitra Modhukur

**Affiliations:** a Integrative Multiomics Lab, School of Bio Sciences and Technology, Vellore Institute of Technology, Vellore, India; b Department of Obstetrics and Gynecology, Institute of Clinical Medicine, University of Tartu, Tartu, Estonia; c Celvia CC AS, Tartu, Estonia; d Division of Obstetrics and Gynecology, Department of Clinical Science, Intervention and Technology (CLINTEC), Karolinska Institute and Karolinska University Hospital, Stockholm, Sweden

**Keywords:** Circulating miRNA, pan-cancer prediction, artificial intelligence, deep learning, SHAP, biomarker discovery, drug repurposing, molecular docking, oncology

## Abstract

Cancer diagnosis at an early stage is crucial for improving overall health outcomes. However, existing cancer diagnostic techniques are mostly invasive and tend to identify the disease only in its advanced stages. MicroRNAs (miRNAs), which are small non-coding RNAs involved in gene expression regulation, are stable in serum as circulating miRNAs and have potential as non-invasive biomarkers. However, their application in pan-cancer diagnostics and therapeutics is still largely unexplored. We developed Serum-MiR-CanPred, a deep learning framework using a multi-layer perceptron (MLP) trained on serum miRNA expression data from 20,271 samples across 12 cancer types and healthy controls from GEO databases. The model achieves robust pan-cancer classification (AUC = 96.87%, accuracy = 96%) with a consensus set of 88 miRNAs. Validation using external datasets demonstrated its generalizability and clinical potential. SHapley Additive exPlanations (SHAP) identified hsa-miR-5100 as a key biomarker, dysregulated in cancers including lung, bladder, and gastric carcinomas. Pathway analysis linked these miRNAs to cancer-related processes like VEGFA-VEGFR2 signalling. Molecular docking of pre-mir-5100 with rDock, identified AC1MMYR2 as a potential high-affinity ligand, with binding stability confirmed by molecular dynamics simulations using GROMACS In conclusion, Serum-MiR-CanPred integrates explainable AI with molecular modelling, advancing miRNA-based diagnostics and drug discovery for precision oncology.

## 1. Introduction

Cancer remains a significant global health issue, causing millions of deaths annually, and is one of the top causes of mortality worldwide [1]. The World Health Organization anticipates a notable increase in new cases by 2040, largely due to ageing populations and lifestyle factors, especially in low-resource areas, where early detection is challenging [2]. Early cancer detection is crucial for enhancing survival rates. For instance, the five-year survival rate for breast cancer detected early and localized is significantly higher than that in cases where the cancer has metastasized. However, several cancer types of progress asymptomatically during early stages, while current diagnostic modalities often fall short of detecting them promptly [2,3].

Traditional diagnostic techniques, such as mammography, endoscopy, and tissue biopsy, are often limited by their invasiveness, high costs, and difficulty in routine screening [4,5]. In contrast, protein-based biomarkers, such as prostate-specific antigen (PSA), have poor specificity, leading to false positives and unnecessary interventions [6,7]. These limitations underscore the urgent need for sensitive, non-invasive, and broadly applicable diagnostic tools capable of detecting multiple cancer types at early stages.

Liquid biopsies using blood-based biomarkers present a promising solution in cancer diagnosis. In particular, circulating microRNAs (miRNAs), small (~22 nucleotides), and non-coding RNAs regulating post-transcriptionally gene expression has emerged as highly attractive biomarker candidates. miRNAs are remarkably stable in blood and other body fluids because of encapsulation in exosomes or binding to proteins, making them well-suited for non-invasive diagnostics [8,9]. These molecules are deeply implicated in cancer-related processes, such as proliferation, apoptosis, and metastasis. The dysregulation of specific miRNAs is common across cancer types. For example, miR-21, an established oncomiR, is overexpressed in breast and colorectal cancers, whereas miR-34a acts as a tumour suppressor and is often downregulated in lung cancer [10,11]. Importantly, certain circulating miRNAs, such as miR-141 and miR-122, are also linked to metastasis and prognosis in prostate and liver cancers, respectively [12,13].

Recent research has highlighted the potential of circulating miRNA signatures to differentiate cancer patients from healthy individuals. For instance, Wu et al. [14] demonstrated that miRNA-pair features can diagnose multiple cancer types with high sensitivity. While, Matsuzaki et al. [15] assembled the large-scale serum miRNA resource GSE211692 and developed the Hierarchical Ensemble Algorithm with Deep Learning (HEAD) classifier, trained on 7,931 cancer and 5,013 non-cancer samples and validated on 1,990 cancer and 1,256 non-cancer samples [15]. In an independent cohort, HEAD achieved 0.88 accuracy for 13-class tissue-of-origin. Notably, the study did not report explicit minority-class oversampling (e.g. SMOTE), an aspect we address here alongside multi-GEO aggregation. Other models using classical ML approaches have shown reduced accuracy in challenging cancer types, such as pancreatic and colorectal cancers [16,17]. Moreover, many prior studies have not explored the full potential of deep learning (DL) architectures, such as Multi-layer Perceptron (MLPs) or Convolutional Neural Networks (CNNs), which can capture non-linear relationships and improve classification performance [18].

At the same time, circulating miRNAs hold therapeutic potential. Their aberrant expression can be pharmacologically modulated using small molecules, antisense oligonucleotides, or RNA mimics. Advances in computational pipelines now allow for the in-silico prediction of drug-miRNA or drug–gene interactions, enabling the repositioning of existing drugs or the discovery of novel modulators [19,20]. Several bioinformatics resources and molecular docking tools now support these analyses, making miRNA-guided drug discovery a feasible direction for ML-powered translational research.

Building upon these insights, in this study we developed Serum-MiR-CanPred, a reproducible artificial intelligence (AI) framework that integrates deep learning and explainable AI for pan-cancer classification using serum miRNAs, while also exploring pharmacological targeting of key miRNAs. Using publicly available data from the Gene Expression Omnibus (GEO), we trained an optimized MLP classifier on 12 cancer types and healthy controls, achieving a robust diagnostic performance with an AUC of 96.87%. The exceptional performance of the model on external validation data sets ensures the generality of the model. Furthermore, to enhance model interpretability, we applied SHapley Additive exPlanations (SHAP) to identify top-contributing miRNAs. Among them, hsa-miR-5100 emerged as a consistent and influential biomarker, being dysregulated in 11 of the 12 cancer types, including breast, prostate, lung, gastric, and hepatocellular carcinomas. This biomarker was further examined in silico for therapeutic relevance through molecular docking using rDock and molecular dynamics simulations with GROMACS, leading to the identification of small molecules, such as AC1MMYR2, that bind to the pre-miRNA Dicer processing site, potentially modulating its expression.

Overall, this study illustrates the dual-purpose approach of diagnosing multiple cancers non-invasively while proposing pharmacological drug targets, marking a significant advancement in precision oncology and bridging computational biology and translational research. By integrating explainable deep learning with molecular docking and drug repurposing, Serum-MiR-CanPred offers a novel and reproducible framework that bridges computational biology and translational medicine. This study aligns with the growing field of ML- and DL-guided cancer pharmacology, where AI accelerates biomarker discovery, target validation, and therapeutic innovations.

## 2. Methodology

### 2.1. Data collection and processing

We obtained gene expression data from the Gene Expression Omnibus (GEO) database under accession numbers GSE113740, GSE164174, GSE211692, and GSE212211 [21]. The data contains miRNA profiles from serum samples of individuals with various cancers and non-cancer controls, measured using the Toray’s 3D-Gene miRNA microarray platform (version 21). The combined dataset included 20,271 samples encompassing 12 cancer types alongside non-cancer samples, as summarized in Table 1 and Supplementary Figure S1.

**Table 1.** Summary of datasets used for pan-cancer classification, including 4 GEO accession numbers, 12 cancer types, healthy controls, and total sample counts.

Cancer Type	GSE113740	GSE164174	GSE211692	GSE212211	Total
Bladder Cancer	25	0	399	0	424
Healthy Controls (No Cancer)	969	1417	5643	0	8029
Breast Cancer	25	0	675	0	700
Biliary Tract Cancer	25	0	402	41	468
Colorectal Cancer	25	50	1596	0	1671
Oesophageal Squamous Cell Cancer	25	50	566	0	641
Gastric Cancer	25	1417	1418	0	2860
Hepatocellular Cancer	345	0	348	383	1076
Lung Cancer	25	0	1699	0	1724
Ovarian Cancer	25	0	400	0	425
Pancreatic Cancer	25	0	851	0	876
Prostate Cancer	25	0	1027	0	1052
Sarcoma	26	0	299	0	325
Total	1590	2934	15323	424	20271

Sample counts reflect raw data from GEO datasets, preprocessed with PyComBat for batch effect correction and SMOTE for class imbalance (Data collection and processing). High Gastric Cancer counts in GSE164174 and GSE211692 result from dataset-specific sampling; no sample overlap was detected. For background subtraction, the mean of the filtered blank signals was subtracted from the sample intensities.

To determine the presence of miRNAs, the sample signals were compared with a threshold established from blank signals. After removing the top and bottom 5% of the blank signal intensities, the threshold was calculated as the mean plus two standard deviations of the remaining values. For the detected miRNAs, the mean of the filtered blank signals was subtracted from the sample intensities. Signals that were not detected were assigned a value of 0.1 on the log2 scale. Data normalization was performed using internal control miRNAs (hsa-miR-149-3p, hsa-miR-2861, and hsa-miR-4463), similar to previous studies [22,23]. Batch effects were adjusted using PyComBat [24], and the dataset was labelled and combined for further analysis.

### 2.2. Data splitting and balancing

The processed miRNA data were divided into training, evaluation, and test sets with a 60:20:20 ratio using the *train_test_split* function from the scikit-learn library [25]. The sample identifiers for the samples in training, evaluation and test set were listed and checked, ensuring no repeats (Supplementary Table 1). StandardScaler was employed to scale the data, while LabelEncoder was used to encode the data labels; the further details are presented in Supplementary Tables −2,3 for reproducible results. To balance the dataset classes, the Synthetic Minority Over-sampling Technique (SMOTE) from the imblearn library was applied to balance the training set [26].

### 2.3. Feature selection

Recursive feature elimination (RFE) from the scikit-learn library was used to select the 100 most important serum miRNAs based on their feature importance from the 1630 total miRNA features. Subsequently, five different estimators, namely Random Forest, Linear Regression, Lasso, LightGBM, Support Vector Machines, were used to select the important features. The features that were common between 3 or more of the top 100 selected features were also compiled and are known as the Consensus Feature Set (CFS) subsequently. These sets of selected features were used to train the different ML and DL models.

### 2.4. Training and comparison of machine learning and deep learning models

To identify the most effective model for pan-cancer prediction, we trained and compared six models across three categories: traditional machine learning, ensemble methods, and deep learning. The characteristics, implementation, and performance considerations of each model are detailed below.

1. k-Nearest Neighbours (kNN): This is a traditional model that makes a prediction by comparing the new data points to the old data points; it relies on the principle that similar data points should have similar classifications. Although simple and fast, KNN tends to become computationally expensive for high-dimensional data [27].
2. Support Vector Machines (SVM): This is a traditional model that computes the optimal multi-dimensional hyperplane to maximize the margin between the data points of different classes. The wide gap between the classes leads to better generalization and prediction accuracy. They become computationally expensive for large datasets and are prone to overfitting [28].
3. Random Forest (RF): An ensemble-based machine learning model that trains multiple decision trees on random subsets of the data and takes the majority vote to make a prediction [29]. It can handle high-dimensional data and resist overfitting while achieving high accuracy.
4. LightGBM: This is a gradient boosting framework that uses decision trees to make predictions. It has a leaf wise tree growth strategy that splits the leaf with the maximum gain, which determines the best splits in the decision trees while pruning the lower gain splits early. It is very fast, even with large datasets, and can handle high dimensional data efficiently [30].
5. Multi-Layer Perceptron (MLP): It is a deep learning model that consists of multiple layers with many interconnected neurons. It has input and output layers with multiple hidden layers that learn the complex patterns in the data, and the weights of the neurons in the hidden layers are adjusted iteratively using backpropagation. The MLP can handle non-linearly separable and high-dimensional data, whereas dropout and cross-validation can be used to prevent overfitting [31].
6. Convolutional Neural Network (CNN): It is a deep learning model that consists of multiple layers, with an input and output layer with convolutional layer (feature extraction), activation layer (learn complex patterns), pooling layer (dimension reduction), flattening layer (convert to 1D vector), and a fully connected dense layer (classification). They can handle high dimensional data and learn from spatial patterns while using dropout to prevent overfitting. We employed the *keras* library from TensorFlow to implement this model [32].

The kNN, SVM, and RF models were implemented using the scikit-learn library [25], the LightGBM model was implemented using lightgbm [30], and the MLP and CNN were implemented using the *keras* library from TensorFlow to implement this model [33]. The hyperparameters for each model were optimized using Optuna for each of the different selected features, and the best hyperparameters were used to train the model [34].

The test set was used to evaluate the optimized models using the standardized metrics described in previous studies [35,36], as follows:

- Accuracy: *(TP + TN]/(TP + TN + FP + FN)*
- Precision: *TP/(TP + FP)*
- Recall: *TP/(TP + FN)*
- F1-score: *2TP/(2TP + FP + FN)*
- Sensitivity: *TP/(TP + FN)*
- Area Under Curve (AUC): The area under the Receiver Operating Characteristic (ROC) curve. The ROC curve is a graphical plot that illustrates the diagnostic ability of a binary classifier by plotting the True Positive Rate (TPR) against the False Positive Rate (FPR) at various threshold settings. AUC measures the ability of a model to distinguish between classes. A higher AUC value indicates better model performance, with a value of 1.0 representing perfect classification and 0.5 indicating no discriminative power.True positives (TP), false positives (FP), true negatives (TN), and false negatives (FN) reflect the model’s correct and incorrect predictions.

The metrics and confusion matrix were calculated using the *classification_report* and *confusion_matrix*. The ROC curve was plotted for each class, and the Area Under the Curve was calculated using the roc_curve and auc functions. The functions used are a part of the scikit-learn library. The best model, MLP with combined important features, was validated using a 5-fold cross-validation approach using the *StratifiedKFold*. The final model was then optimized, trained, and saved for future applications.

### 2.5. External validation of the model

External validation was performed using additional GEO datasets that employed the same Toray’s 3D-Gene miRNA microarray platform for measuring serum miRNA expression in cancer samples. Specifically, three GEO datasets with accession IDs GSE106817, GSE112264 and GSE124158 were utilized to encompass all cancer types relevant to our model, the sample identifiers for the datasets can be found in Supplementary Table 1. The optimal model was also trained on the original dataset without the application of SMOTE, thereby eliminating potential artefacts from synthetic samples. To prevent data overlap, any duplicates between the validation set and the original dataset were removed. The dataset underwent minimal pre-processing, including blank correction, normalization based on internal controls, and Z-score transformation. The preserved model was then validated on these datasets, and the metrics and AUC were calculated accordingly.

### 2.6. Data visualization and interpretability

A heatmap of miRNA expression in CFS across cancer types was generated using the heatmap function from Seaborn, revealing patterns of miRNA overexpression and underexpression across cancer types [37]. Dimensionality reduction techniques, Principal Component Analysis (PCA) and t-distributed Stochastic Neighbor Embedding (t-SNE), were applied using scikit-learn’s *pca* and *tsne* functions to visualize the separation between cancer and non-cancer samples. Feature importance was assessed using SHAP (SHapley Additive exPlanations), with the *summary_plot* function generating beeswarm plots to highlight the top miRNAs contributing to the predictions for each cancer type [38].

### 2.7. In silico functional validation

Validated gene targets for the CFS miRNAs were identified using the get_multimir function from the multiMiR library in R, cross-referencing the miRecords, miRTarBase, and TarBase databases, with targets present in at least two databases selected for analysis [39]. Pathway enrichment was performed using Metascape to generate bar plots and network graphs of significant pathways and ontology clusters [40]. KEGG pathway analysis was conducted using the g:Profiler webserver, and the results visualized using ggplot2 in R [41].

### 2.8. RNA-targeted drug discovery

For therapeutic exploration, hsa-miR-5100 was selected based on its top SHAP importance score and evidence of overexpression across cancer types [42]. Small molecule inhibitors were identified from literature, where compounds were reported to inhibit Dicer-mediated processing of pre-miRNA, reducing expression of the corresponding mature target miRNA. Some molecules are approved drugs, while others are novel compounds proposed by the authors. The pre-miRNA sequence and secondary structure were retrieved from miRBase (v22.1) [43]. RNAComposer created a three-dimensional model by applying energy minimization and secondary structure constraints, using default settings of 100 iterations and a 0.1 kcal/mol threshold [44]. RNAsite identified binding sites on pre-miR-5100, with a focus on the Dicer cleavage site due to its significance in miRNA maturation and its potential for therapeutic intervention [45,46]. The SMILES representations of the small molecules were obtained using Marvin JS from ChemAxon (https://www.chemaxon.com), and their corresponding 3-D structures were retrieved from PubChem using these SMILES strings. Next, molecular docking was performed using rDock, screening the small molecules collected from literature [47]. The rDock master scoring function, which is a weighted sum of intermolecular, ligand intramolecular, site intramolecular, and external restraint terms, was used to evaluate the docking of small molecules with pre-miRNA. Docking results involving pre-miR-5100 were compared with those obtained for other pre-miRNAs previously validated in the literature. The results of docking were further assessed using negative controls for the best ligand. Specifically, random miRNAs were chosen and docked with the best ligand. The binding affinity and hydrogen bonds were compared with the hsa-mir-5100 and ligand complex.

Molecular dynamics simulations used GROMACS (v2020.2) with the AMBER OL3 force field for RNA [48,49]. The pre-hsa-miR-5100-AC1MMYR2 complex was solvated in a cubic box (TIP3P water model), neutralized with Na^+^/Cl^−^ ions, and equilibrated under NVT (300 K, 100 ps) and NPT (1 bar, 100 ps) ensembles. A 100-ns production run assessed stability via root mean square deviation (RMSD), root mean square fluctuation (RMSF), radius of gyration (Rg), and hydrogen bond occupancy, compared to hsa-miR-21 as a control [48, 50–53].

## 3. Results

### 3.1. Overview of analytical steps

**Figure 1.**
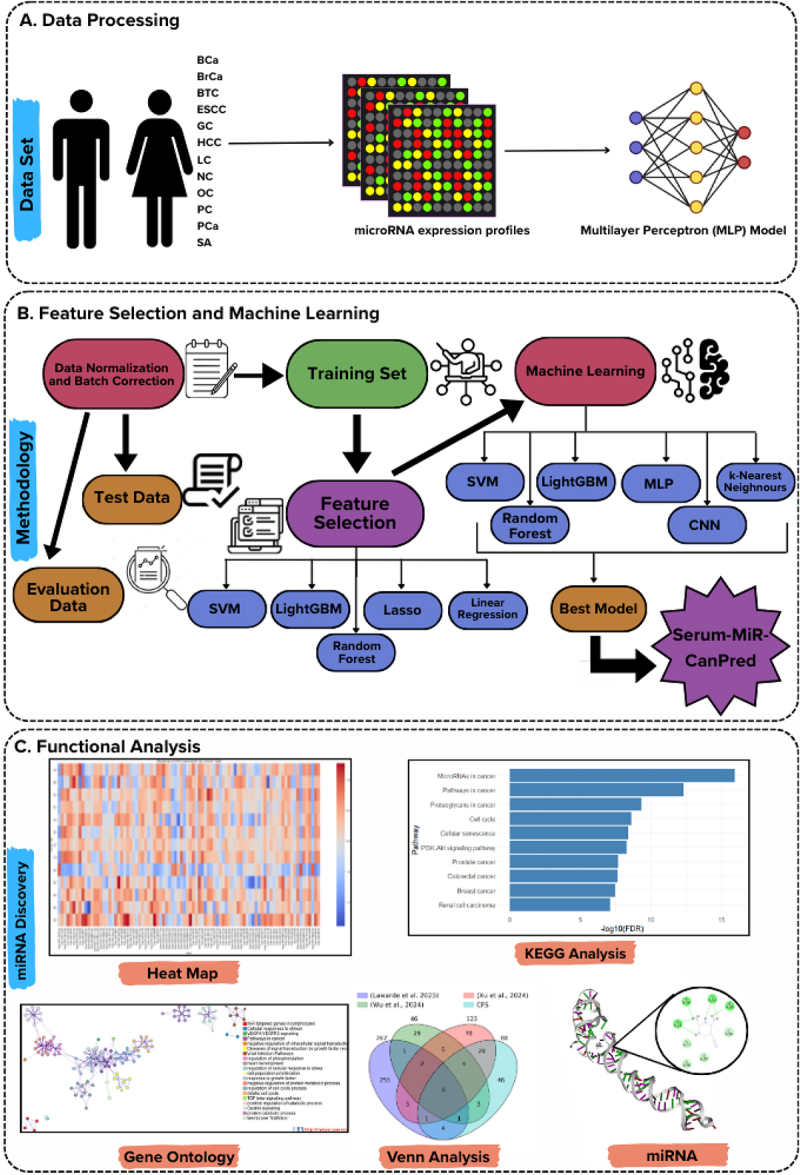
Overview of the machine learning framework for pan-cancer classification implemented in this study and therapeutic exploration. The workflow includes (A) data processing with preprocessing, (B) feature selection and machine learning with SHAP, and (C) functional analysis including docking.

A structured analytical pipeline was created to classify tumours using miRNA expression originating from serum samples, as shown in Figure 1. Datasets from the GEO, containing 20,271 samples, underwent normalization and batch effect correction. The data were split into training (60%), evaluation (20%), and test (20%) subsets. The minority classes in the training set were up-sampled to create a balanced dataset, resulting in 4,817 samples per class and 62,621 training samples. Feature selection used five algorithms: SVM, LightGBM, Lasso, Linear Regression, and Random Forest, each identifying the top 100 important features. The features common between the selected features formed a Consensus Feature Set (CFS, *n* = 88), reducing dimensionality while maintaining biological significance. Classification algorithms, including SVM, LightGBM, MLP, KNN, Random Forest, and CNN, were trained with the selected features and optimized using Optuna for hyperparameter tuning. The best model–feature combination was selected based on the evaluation set performance. The final model was validated using an independent test set and through 5-fold cross-validation. SHAP analysis was used to assess feature importance, and enrichment analyses using GO, KEGG pathways, and gene annotation databases were used to evaluate functional significance. Molecular docking and molecular dynamics simulations identified small molecules that interact with miRNAs to inhibit their expression. The detailed results are described in subsequent sections.

### 3.2. Feature selection

The top 100 most important features were chosen using Recursive Feature Elimination (RFE) with five different estimators. There were 88 miRNAs in CFS. The list of important miRNAs can be found in Supplementary Table −4.

### 3.3. Comparison of models

The MLP models had the highest overall metrics with 94% accuracy, and the classification reports were compared for the top models, and the MLP model trained on the CFS was established as the best model. The overall metrics for the models are listed in Table 2 and the classification reports are listed in Supplementary Table 5. The model trained without SMOTE had a 2% lower accuracy and the precision was significantly less for some cancers like biliary tract and ovarian cancer. The complete classification matrix can be found as Supplementary Table −6.

**Table 2.** Performance metrics of machine learning models trained on selected features for pan-cancer classification.

Model	Feature Selection	Feature Count	Accuracy	Precision	Recall	F1-Score
Multi-Layer Perceptron	CFS	88	0.94	0.94	0.94	0.94
Multi-Layer Perceptron	Lasso	100	0.94	0.94	0.94	0.94
Multi-Layer Perceptron	LightGBM	100	0.94	0.94	0.94	0.94
Multi-Layer Perceptron	Logistic Regression	100	0.94	0.94	0.94	0.94
Multi-Layer Perceptron	SVM	100	0.93	0.93	0.93	0.93
SVM	CFS	88	0.93	0.93	0.93	0.93
SVM	Lasso	100	0.93	0.93	0.93	0.93
SVM	LightGBM	100	0.93	0.93	0.93	0.93
Multi-Layer Perceptron	Random Forest	100	0.92	0.92	0.92	0.92
SVM	Logistic Regression	100	0.92	0.92	0.92	0.92
Convolutional Neural Network	CFS	88	0.91	0.91	0.91	0.91
Convolutional Neural Network	Lasso	100	0.91	0.91	0.91	0.91
Convolutional Neural Network	Random Forest	100	0.91	0.91	0.91	0.91
Convolutional Neural Network	SVM	100	0.91	0.91	0.91	0.91
LightGBM	CFS	88	0.91	0.91	0.91	0.91
LightGBM	Lasso	100	0.91	0.91	0.91	0.91
LightGBM	Random Forest	100	0.91	0.91	0.91	0.91
SVM	Random Forest	100	0.91	0.91	0.91	0.91
SVM	SVM	100	0.91	0.91	0.91	0.91
Convolutional Neural Network	LightGBM	100	0.9	0.9	0.9	0.9
Convolutional Neural Network	Logistic Regression	100	0.9	0.91	0.9	0.9
LightGBM	Logistic Regression	100	0.89	0.89	0.89	0.89
Random Forest	CFS	88	0.89	0.89	0.89	0.89
Random Forest	Lasso	100	0.89	0.89	0.89	0.89
Random Forest	LightGBM	100	0.89	0.9	0.89	0.89
K-Nearest Neighbours	LightGBM	100	0.88	0.88	0.88	0.88
LightGBM	LightGBM	100	0.88	0.89	0.88	0.89
Random Forest	Logistic Regression	100	0.88	0.88	0.88	0.88
Random Forest	Random Forest	100	0.88	0.88	0.88	0.88
Random Forest	SVM	100	0.88	0.88	0.88	0.87
K-Nearest Neighbours	CFS	88	0.87	0.87	0.87	0.87
LightGBM	SVM	100	0.87	0.88	0.87	0.87
K-Nearest Neighbours	Lasso	100	0.86	0.86	0.86	0.86
K-Nearest Neighbours	Random Forest	100	0.86	0.86	0.86	0.86
K-Nearest Neighbours	Logistic Regression	100	0.84	0.85	0.84	0.84
K-Nearest Neighbours	SVM	100	0.84	0.85	0.84	0.84

**Figure 2.**
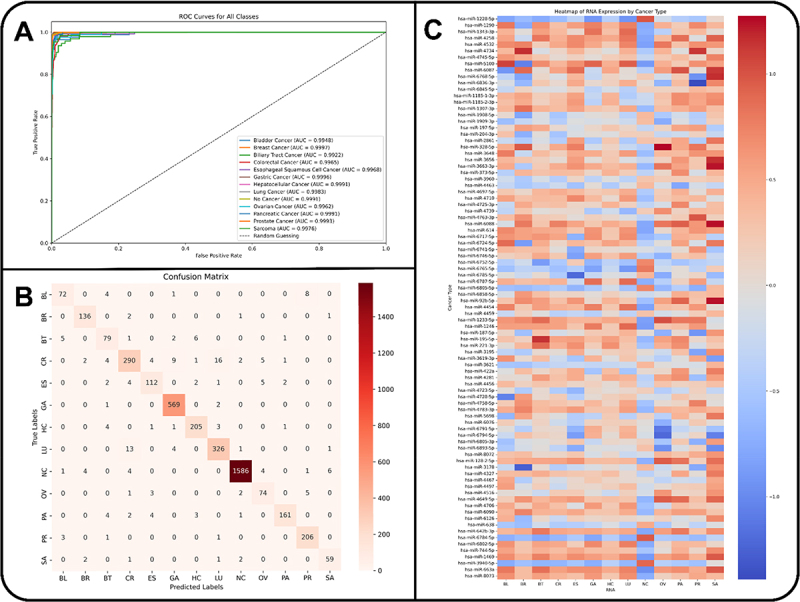
Performance evaluation of serum-MiR-CanPred. (A) receiver Operating characteristic (ROC) curves for each class, demonstrating the model’s ability to distinguish between them. Different colors represent various classes, with their respective Area under the curve (AUC) values shown in the legend. (B) confusion matrix for the serum-MiR-CanPred model showing classification outcomes for all classes. Rows indicate actual labels, while columns show predicted labels. The intensity of the color corresponds to the number of predictions, with darker shades signifying higher counts. (C) heatmap illustrating the correlation between miRnas in the CFS list and different cancer types. The color intensity indicates the correlation coefficient, ranging from −1 (blue, indicating a negative correlation) to 1 (red, indicating a positive correlation), with a gradient scale provided on the right.

The MLP-CFS model during the 5-fold cross-validation had a consistent accuracy of 95%. The final model trained on the best hyperparameters had an overall accuracy of 96%, the precision of all classes was greater than 80% with a weighted average of 96%, the recall of all classes was greater than 84% with a weighted average of 96%, the f1-score of all classes was greater than 82% with a weighted average of 96%, the sensitivity of all classes was greater than 99%, and the AUC score for all classes was greater than 0.99 (Table 3, Figure 2(A)). The confusion map of the best model on the test set is shown in Figure 2(B). This model is hereafter referred to as the *Serum-MiR-CanPred*.

**Table 3.** Performance metrics of the serum-MiR-CanPred model (MLP trained on CFS) for 12 cancer types and healthy controls.

	Precision	Recall	F1-Score	Specificity	AUC Score
Bladder Cancer	88.89%	84.71%	86.75%	99.77%	0.9949
Breast Cancer	94.44%	97.14%	95.77%	99.80%	0.9997
Biliary Tract Cancer	79.80%	84.04%	81.87%	99.50%	0.9922
Colorectal Cancer	90.94%	87.13%	88.99%	99.22%	0.9965
Oesophageal Squamous Cell Cancer	89.06%	89.06%	89.06%	99.64%	0.9967
Gastric Cancer	97.09%	99.13%	98.10%	99.51%	0.9997
Hepatocellular Cancer	95.35%	95.35%	95.35%	99.74%	0.9991
Lung Cancer	92.88%	94.49%	93.68%	99.33%	0.9984
Non-Cancer	99.44%	98.69%	99.06%	99.63%	0.9991
Ovarian Cancer	84.09%	87.06%	85.55%	99.65%	0.9961
Pancreatic Cancer	97.56%	91.43%	94.40%	99.90%	0.9992
Prostate Cancer	93.64%	97.63%	95.59%	99.64%	0.9994
Sarcoma	88.06%	90.77%	89.39%	99.80%	0.9976

### 3.4. External validation of the model

The datasets utilized for external validation yielded promising outcomes. Specifically, the model achieved an average AUC score of 99% and a precision of 91% for the GSE124158 dataset, which includes the classes BR, CR, ES, GA, HC, LU, PA, and healthy controls. In the case of the GSE112264 dataset, which predominantly consists of Prostate cancer samples, the precision was 99% with an AUC score of 99%, while the other classes had an average AUC of 94.27%. For the GSE106817 dataset, the average accuracy was 89%, with an average AUC of 99.03%. Collectively, these databases encompass all the cancer classes examined in our study, demonstrating the model’s capability to utilize miRNA expression data. The class wise metrics for all three validation datasets is shown in Supplementary Table 7.

### 3.5. Analysis of the miRNA in CFS

The heatmap of the expression levels of the 88 miRNAs in CFS across the cancer types revealed the overexpressed and under-expressed miRNAs for the different types (Figure 2(C)). Dimensionality reduction techniques were applied to visualize the global patterns of RNA expression. Both PCA and t-SNE demonstrated a separation between non-cancer (NC) and cancer samples. In the PCA plot (Suplementary Figure S2), NC samples clustered tightly and distinctly, whereas cancer samples formed a more dispersed cluster with substantial overlap between different cancer types. Similarly, the t-SNE plot (Suplementary Figure S3) showed a well-defined cluster of NC samples, whereas cancer types were intermixed, forming a broad but coherent cluster. These results suggest that while non-cancer and cancer samples are easily distinguishable, individual cancer types share similar expression profiles, and non-linear methods are required to separate them.

**Figure 3.**
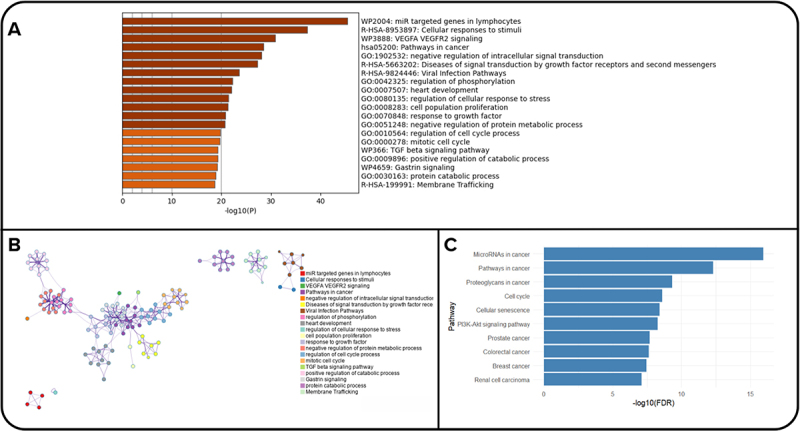
Functional enrichment analysis of miRNA targets. (A) bar plot showing top GO biological processes enriched among validated targets of selected miRnas using metascape. (B) network-based visualization of clustered GO terms and pathways, with color-coded modules representing related biological themes. (C) KEGG pathway enrichment results highlighting associations between miRNA targets and known cancer-related conditions using g:profiler. Significance is reported as -log10(FDR).

A total of 1102 validated gene targets were extracted from the *miRecords*, *miRTarBase*, and *TargetScan* databases (Supplementary Table 8). Metascape analysis revealed that the top three pathways for the extracted gene targets were miR-targeted genes in lymphocytes, cellular responses to stimuli, and VEGFA VEGFR2 signalling are the top three pathways for the extracted gene targets (Figure 3(A)). The enriched ontology cluster of the genes revealed the interconnection of the genes involved in different pathways (Figure 3(B)). KEGG analysis revealed that the most significant pathways were related to cancer and cell cycle pathways (Figure 3(C)).

### 3.6. Analysis of the MLP model

**Figure 4.**
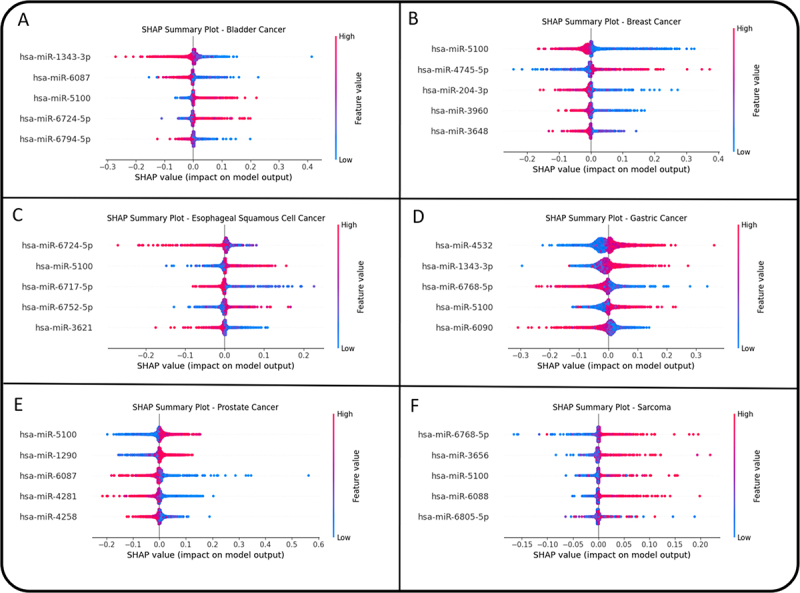
SHAP summary plots illustrating the impact of top miRNA features on cancer-type classification models. (A–F) each panel shows the SHAP values for the top five miRnas in bladder, breast, esophageal squamous cell, gastric, prostate, and sarcoma cancers, respectively. Dots represent individual SHAP values for each sample; color indicates the feature (miRNA) expression value (red = high, blue = low). Features are ranked by their average absolute impact on model output.

The feature importances for miRNAs were calculated using SHAP for the model, and the beeswarm plots show the top five miRNA contributions to the prediction of each cancer type (Figure 4, Supplementary Figure S4). The plots revealed the differential expression of miRNAs in different cancer types.

### 3.7. Comparison of CFS with similar pan-cancer studies

To evaluate the relevance of our selected candidate feature set (CFS), we compared it with serum miRNA sets reported in recent studies (Supplementary Figure S5, Supplementary Table S9). Wu et al. [14] identified 46 important miRNAs using LASSO regression, while Xu et al. [17] used RFE with a LASSO estimator to select 123 miRNAs. Lawarde et al. [54] compiled a list of 267 miRNAs that are differentially expressed in cancers. Our CFS consists of 88 miRNAs. Of these, 29 overlapped with Xu et al., 3 with Wu et al., and 4 with both Xu et al. and Wu et al. Additionally, 4 miRNAs overlapped with Lawarde et al.‘s list, and 1 overlapped with both Wu et al. and Lawarde et al. These overlaps demonstrate both consistency with prior computational approaches and the potential novelty of our feature selection strategy.

### 3.8. Selection of candidate miRNA for targeted drug discovery

hsa-miR-5100 was selected for targeted drug discovery because of its importance in predicting various types of cancer. According to the SHAP analysis, hsa-mir-5100 was among the top five most important features for bladder cancer, breast cancer, gastric cancer, oesophageal squamous cell cancer, pancreatic cancer, prostate cancer, and sarcoma. The box plot with the median expression revealed overexpression of hsa-mir-5100 across all cancer types except breast cancer (Supplementary Figure S6).

### 3.9. RNA-targeted drug discovery: pre-miR-5100 as a case study

**Figure 5.**
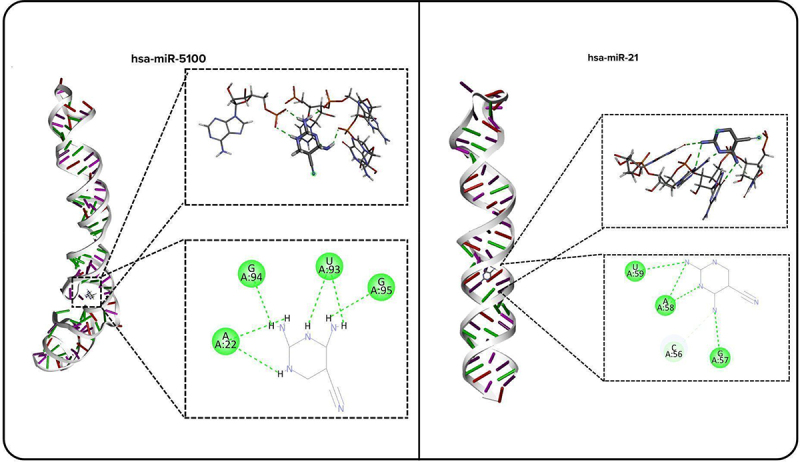
Binding of AC1MMYR2 to hsa-miR-5100 and hsa-miR-21. (A) interaction of AC1MMYR2 with hsa-miR-5100. (B) interaction between AC1MMYR2 and hsa-miR-21. 3D (top) and 2D (bottom) views highlight key binding residues, hydrogen bonds (green dashed lines) and carbon-hydrogen bonds (light green dashed lines).

We explored the therapeutic potential of hsa-miR-5100, identified via SHAP as a key biomarker, using molecular docking with rDock and dynamics simulations with GROMACS. hsa-miR-5100, identified through SHAP analysis as a top pan-cancer biomarker, was prioritized for therapeutic targeting due to its overexpression and regulatory involvement across several cancer types [45,55]. A virtual screening was conducted on potential small molecules, identified in published literature, that are known to target pre-miRNA using rDock (Table 4). Among the screened compounds, AC1MMYR2 (PubChem CID: 3,246,569) exhibited the most favourable binding profile, with a docking score of −68.53, forming seven hydrogen bonds with the Dicer cleavage site of pre-hsa-miR-5100, outperforming known interaction with pre-hsa-miR-21 (−36.251, 4 hydrogen bonds) (Table 4). Key interactions were observed with nucleotides A22, U93, G94, and G95, which formed stable bonds with pre-miR-5100, inhibiting Dicer binding (Figure 5). AC1MMYR2 adheres to Lipinski’s rule of five (MW = 141.1 g/mol, logP = −0.817, hydrogen bond donors = 4), suggesting favourable drug-likeness [56].

**Table 4.** Docking scores of small molecules with hsa-miR-5100 compared to controls, including hydrogen bond counts.

Pubchem ID	Small Molecule Name	Proven target miRNA	Docking Score	Control Score	H-Bond in mir-5100	H-Bond in Complex	Reference
3246569	AC1MMYR2	pre-miR-21	−68.5257	−36.251	6	4	Shi et al. [53]
445154	Resveratrol	pre-miR-21	−65.2039	−28.5719	3	6	Sheth et al. [58]
4212	mitoxantrone	pre-miR-21	−61.0837	−28.8339	7	7	Velagapudi et al. [59]
46190600	Modified azobenzene	pre-miR-21	−59.6447	−26.3629	1	1	Gumireddy et al. [60]
19649	Streptomycin	pre-miR-21	−53.6812	−44.8209	14	9	Bose et al. [61]
44221088	Targapremir-210	pre-miR-210	−51.9284	−53.6709	1	4	Costales et al. [62]
166778834	Targapremir-18a	miR-18a	−38.5336	−24.2897	1	0	Velagapudi et al. [63]
72699186	MIR96-IN-1	pre-miR-96	−37.8321	44.6072	1	0	Velagapudi et al. [64]

**Figure 6.**
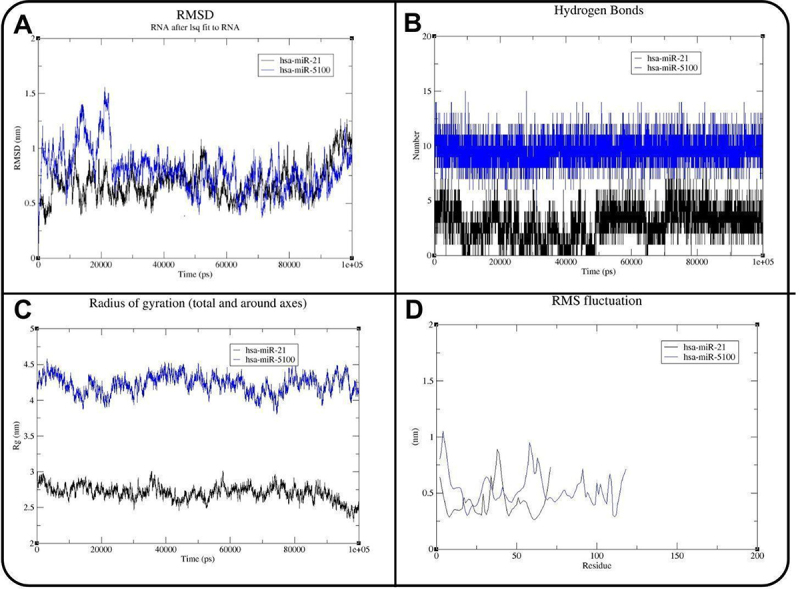
Molecular dynamics analysis of hsa-miR-21 (black) and hsa-miR-5100 (blue) complexes with AC1MMYR2. (A) RMSD of RNA backbones over time (x-axis: time in ps; y-axis: RMSD in nm) showing structural stability. (B) hydrogen bonds formed between RNA and ligand (x-axis: time in ps; y-axis: number of hydrogen bonds) indicating interaction consistency. (C) radius of gyration (x-axis: time in ps; y-axis: Rg in nm) reflecting the structural compactness of each complex. (D) RMSF per residue (x-axis: residue index; y-axis: RMSF in nm) illustrates the nucleotide flexibility throughout the simulation.

To evaluate the stability and specificity of the interaction, molecular dynamics (MD) simulations were conducted using GROMACS for 100 ns, comparing the pre-hsa-miR-5100–AC1MMYR2 complex to a control complex with pre-hsa-miR-21. The root mean square deviation (RMSD) analysis demonstrated that the hsa-miR-5100–AC1MMYR2 complex stabilized over the simulation time, indicating a stable binding conformation (Figure 6(A)). Root mean square fluctuation (RMSF) analysis revealed minimal fluctuations at the binding site, further supporting the stability of the interaction (Figure 6(B)). The radius of gyration (Rg) averaged 4.22 nm for hsa-miR-5100, compared to 2.70 nm for hsa-miR-21, reflecting differences in structural compactness (Figure 6(C)). Furthermore, hydrogen bond occupancy averaged 10 ± 1 interactions for the hsa-miR-5100 complex versus 4 ± 1 for the control, indicating both high specificity and sustained binding affinity (Figure 6(D)). These results collectively support AC1MMYR2 as a promising modulator of hsa-miR-5100 and justify further experimental validation via luciferase reporter assays and in vitro functional studies [57]. To validate the docking process, AC1MMYR2 was also docked with eight random miRNAs as negative controls. The reduced binding affinity in these random miRNAs suggests AC1MMYR2 docking is specific to hsa-miR-5100 (Table 5).

**Table 5.** Docking scores of random miRnas to AC1MMYR2, including hydrogen bond counts.

Sr. No.	miRNA	Docking Score	Hydrogen Bonds
1	hsa-mir-29b	−27.2721	4
2	hsa-mir-614	−27.7591	3
3	hsa-mir-1255a	−30.1346	5
4	hsa-mir-3928	−27.6875	5
5	hsa-mir-4742	−31.1404	4
6	hsa-mir-4745	−23.4815	5
7	hsa-mir-6799	−29.344	5
8	hsa-mir-6870	−19.3396	3

## 4. Discussion and conclusion

This study presents Serum-MiR-CanPred, a deep learning-based framework utilizing an MLP model for pan-cancer classification using 88 circulating miRNAs. The model achieved high diagnostic performance (AUC = 96.87%, accuracy = 96%) across 12 cancer types. Beyond diagnostic classification, the framework integrates explainable AI via SHAP analysis to identify cancer-specific miRNA biomarkers and evaluates their therapeutic relevance using molecular docking and molecular dynamics simulations. Circulating miRNAs have emerged as robust, non-invasive biomarkers for early cancer detection due to their stability in biofluids and cancer-specific expression patterns. While prior studies have predominantly employed tree-based models such as Random Forest, LightGBM, and SVM [20,35,65], these approaches often underperform in complex malignancies like pancreatic cancer. Neural networks, though applied in isolated cases, have not been comprehensively studied for pan-cancer prediction using serum-derived miRNA data [66]. A recent study compared multiple machine learning models, including MLP, using the R package tidymodels. However, the approach lacked the scalability and optimization flexibility provided by TensorFlow [17]. In contrast, Serum-MiR-CanPred employs a well-optimized MLP model implemented in TensorFlow, leveraging its extensive hyperparameter tuning capabilities and architectural customization.

In comparison to the HEAD model introduced by Matsuzaki et al. on the GSE211692 serum miRNA cohort, they reported 13-class tissue-of-origin accuracy of 0.88 overall (0.90 in early stages). Furthermore, the HEAD model achieved an overall multiclass F1 score of 0.93, along with high per-cancer AUCs for distinguishing between cancer and non-cancer cases [15]. In our setting, Serum-MiR-CanPred achieved 0.96 accuracy and a 0.96 weighted F1 across 12 cancers plus controls, with a macro-average AUC of 0.99 under a one-versus-rest evaluation. Although preprocessing pipelines, class definitions, and validation protocols are not identical, the inclusion of explicit class-imbalance handling, multi-GEO data aggregation, and a compact 88-miRNA consensus feature set improves performance, particularly for under-represented cancers, while maintaining state-of-the-art aggregate accuracy and generalizability.

The CFS, consisting of a minimal set of miRNAs (88 miRNAs), was compared with feature sets from similar pan-cancer studies. Wu et al. [14] used LASSO regression to select a set of important miRNAs, while Xu et al. [17] employed recursive feature elimination with LASSO, resulting in a large feature set. In contrast, the CFS in our studies is notably smaller, demonstrating that a minimal set of miRNAs can achieve comparable or superior predictive performance, highlighting the efficiency of our feature selection and model optimization strategies. Overlaps with prior sets (e.g. 29 miRNAs with Xu et al.) validate our approach, while unique miRNAs suggest novel biomarkers.

A notable feature of our study is the inclusion of an external validation set, which enhances the model’s generalizability, as shown by AUCs of 99.03%, 99.62%, and 94.27% on GSE106817, GSE124158, and GSE112264, respectively. The model’s outstanding performance across these GEO datasets, achieved with minimal preprocessing, underscores its potential applicability to clinical samples for cancer type prediction. Moreover, CFS consisting of only 88 miRNAs, with precomputed scaler and labeller values, enables efficient and scalable prediction pipeline from raw data.

Pathway analysis of validated gene targets extracted from databases (miRecords, miRTarBase, and TarBase) provided deeper insights into the biological roles of these miRNAs. Metascape analysis identified key pathways, including lymphocyte activation, immune regulation, cell cycle progression, cellular responses to stimuli, and VEGFA-VEGFR2 signalling, as the most significant for miRNA-targeted genes. Enriched ontology clusters revealed interconnections among these genes across pathways, while KEGG analysis highlighted cancer and cell cycle pathways as the most prominent, underscoring the oncogenic relevance of the identified miRNAs. Additionally, a heatmap of miRNA expression levels in the Consensus Feature Set (CFS) across cancer types revealed distinct patterns of overexpression and underexpression, further supporting their diagnostic potential. PCA and t-SNE visualizations demonstrated a clear segregation between cancer and non-cancer samples, although cancer types clustered together, indicating shared miRNA signatures across malignancies, underscores the model’s diagnostic values and pan-cancer utility. These findings align with miRNA-driven oncogenesis, informing precision oncology.

Another notable feature of this study is the use of SHAP analysis to identify critical miRNAs contributing to cancer prediction, an approach rarely applied in previous miRNA-based studies [38]. SHAP analysis revealed several dysregulated miRNAs with significant roles in various cancers. This approach identified several miRNAs with strong predictive power and biological relevance. For instance, hsa-miR-1343-3p has been linked to bladder cancer [67], hsa-miR-663a has been shown to promote gallbladder cancer by downregulating EMP3 [68], hsa-miR-6090 is overexpressed in colorectal cancer [69], and hsa-miR-4532 is upregulated in gastric cancer [70] consistent with experimental reports. SHAP also revealed subtype-specific miRNAs such as hsa-miR-1233-5p (biliary and hepatocellular cancers), hsa-miR-614 (ovarian), hsa-miR-3656 (pancreatic), and hsa-miR-6768-5p (sarcoma). This interpretability supports both biomarker discovery and future translational application.

In our analysis, hsa-miR-5100 emerged as the top-ranked biomarker, implicated in several malignancies including bladder, breast, gastric, pancreatic, and sarcoma [19,42,67,71,72]. Previous literature describes its oncogenic role in gastric cancer via DEK/AMPK/mTOR signalling [55] and tumour-suppressive activity in prostate cancer through E2F7 targeting [42], reflecting its context-dependent behaviour. Moreover, SHAP analysis in our study also ranked hsa-miR-5100 as a key contributor to pan-cancer classification. However, according to the MirGene database [73], hsa-miR-5100 does not fulfill all the criteria to be recognized as a miRNA, suggesting it may be another type of small RNA or potentially an artefact. Nonetheless, several studies have referred to it as a miRNA, underscoring the need for cautious interpretation and further experimental validation is warranted to confirm its molecular identity and functional roles in cancer progression.

In a case study exploring therapeutic possibilities, we examined the potential of hsa-miR-5100 as a target for drug development. Utilizing rDock, we identified AC1MMYR2 (PubChem CID: 3,246,569) as the most promising ligand, demonstrating strong binding at the Dicer cleavage site [53]. The compound satisfies Lipinski’s rule of five, suggesting drug-likeness [56]. The docking of AC1MMYR2 was validated using random miRNA as negative control [74]. Docking validation with random pre-miRNAs as negative controls revealed significantly lower binding affinities compared to pre-hsa-miR-21 and pre-hsa-miR-5100, with AC1MMYR2 showing stronger affinity for pre-hsa-miR-5100 than for the positive control pre-hsa-miR-21. Subsequent molecular dynamic simulations using GROMACS confirmed stable interactions between AC1MMYR2 and pre-miR-5100, with favourable RMSD, RMSF, and hydrogen bonding profiles compared to a control miRNA (pre-hsa-miR-21) [75,76]. These in silico findings align with current strategies targeting non-coding RNAs targeted drug discovery [51,57,77], though experimental validation is needed to establish hsa-miR-5100’s therapeutic potential, e.g. luciferase assays and cell-based functional studies is warranted.

Despite its strengths, this study has limitations. First, the use of public GEO datasets may introduce technical heterogeneity despite batch correction via PyComBat. Next, the absence of clinical metadata (e.g. tumour stage, grade, patient age, and sex) restricts contextual interpretation. Moreover, serum-based profiles may differ from plasma or whole blood, affecting reproducibility. Finally, while molecular docking and MD simulations offer preliminary therapeutic insights; however, they require in-vitro experimental validation for further clinical insights. Thus, future studies should incorporate external validation cohorts, longitudinal samples, and clinical covariates to improve the model’s translational applicability [78–81].

In conclusion, we developed Serum-MiR-CanPred, a multi-layer perceptron model for the classification of 12 cancer types using a small set of circulating miRNAs. The model demonstrated high diagnostic accuracy (> 95%), offering a non-invasive and efficient approach for multi-cancer detection. The model’s robustness and generalizability were supported by external validation on an independent dataset, maintaining comparable predictive performance. SHAP analysis identified key miRNAs, including hsa-miR-5100 and hsa-miR-1228-5p, which were strongly associated with various cancer types. Functional enrichment analysis further confirmed their involvement in critical cancer-related pathways, such as VEGFA-VEGFR2 signalling and immune regulation. Molecular docking and simulation analyses revealed the therapeutic potential of AC1MMYR2 in downregulating hsa-miR-5100 through stable interactions with known inhibitors. Additionally, docking analyses with negative controls showed no significant binding, thereby strengthening the specificity of the observed drug – miRNA interactions. Overall, the study highlights the diagnostic and therapeutic relevance of circulating miRNAs in cancer classification and treatment.

## Supplementary Material

Supplementary Material.docx

## Data Availability

Publicly available datasets were analysed in this study. This data can be found here: https://www.ncbi.nlm.nih.gov/geo/. The model developed in this study is available from https://huggingface.co/naisarg14/MLP-PanCanPred and the code developed in this study is available at https://github.com/naisarg14/cancer-miRNA
