# Supplementary material for: Serum-MiR-CanPred: deep learning framework for pan-cancer classification and miRNA-targeted drug discovery
Source: RNA Biol. 2025 Oct 30;22(1):1–19. doi: 10.1080/15476286.2025.2577433 (PMC12578308; doi:10.1080/15476286.2025.2577433)
Supplement: Supplementary Material.docx [file KRNB_A_2577433_SM0683.docx]

Supplementary Material

**Serum-MiR-CanPred: Deep Learning Framework for Pan-Cancer Classification and miRNA-Targeted Drug Discovery**

Naisarg Patel^1^, Ankita Lawarde^2,3^, Suhas Manikant Surisetti^1^, Premkumar Thiruselvam^1^, Prakash Lingasamy^1,2^, Vino Sundararajan^1^, Sajitha Lulu. S^1^, Andres Salumets^2,3,4*^, Vijayachitra Modhukur ^2,3*^

1. Integrative Multiomics Lab, School of Bio Sciences and Technology, Vellore Institute of Technology, Vellore – 632014, Tamil Nadu, India
2. Department of Obstetrics and Gynecology, Institute of Clinical Medicine, University of Tartu, L. Puusepa 8, 50406 Tartu, Estonia
3. Celvia CC AS, 50411 Tartu, Estonia
4. Division of Obstetrics and Gynecology, Department of Clinical Science, Intervention and Technology (CLINTEC), Karolinska Institute, and Karolinska University Hospital, Stockholm, Sweden

* To whom correspondence should be addressed.

Dr. Vijayachitra Modhukur, Department of Obstetrics and Gynaecology, Institute of Clinical Medicine, University of Tartu, 50406 Tartu, Estonia. E-mail: [vijayachitra.modhukur@ut.ee](mailto:vijayachitra.modhukur@ut.ee)

* Correspondence may also be addressed to

Prof. Andres Salumets, Division of Obstetrics and Gynaecology, Department of Clinical Science, Intervention and Technology (CLINTEC), Karolinska Institutet, and Karolinska University Hospital, 14152, Stockholm, Sweden. E-mail: [andres.salumets@ki.se](mailto:andres.salumets@ki.se)

1. **Supplementary Figures**


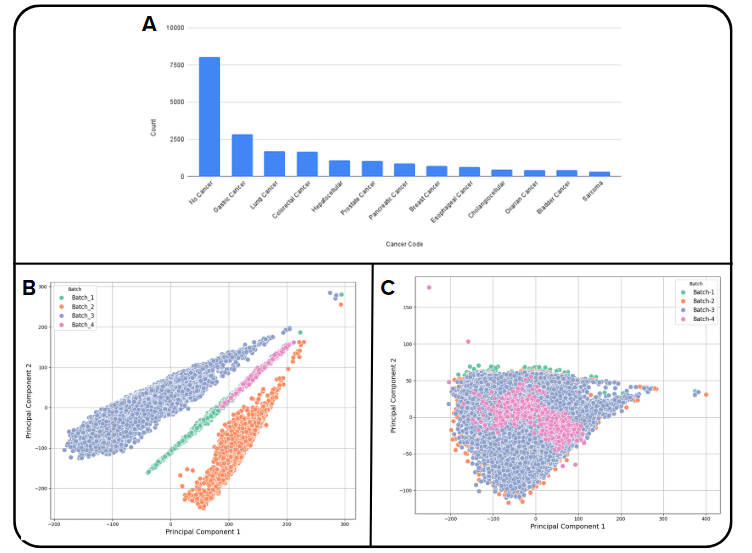


**Supplementary Figure 1.** Overview of dataset and batch effect correction. (A) Distribution of samples across cancer types. (B) Principal Component Analysis (PCA) plot showing clear separation of samples by batch before correction, indicating strong batch effects. (C) PCA plot after batch correction showing successful mixing of samples from different batches, suggesting effective removal of batch effects.


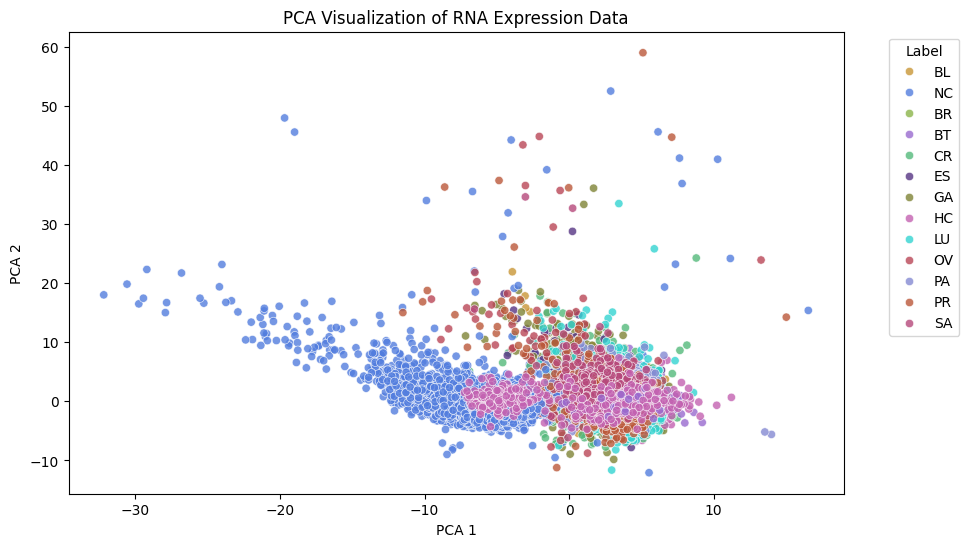


**Supplementary Figure 2.** Principal Component Analysis (PCA) plot of RNA expression data based on the 88 selected features from Consensus Feature Set (CFS). Samples are colored by their respective labels, showing separation patterns across different cancer types and control (NC) groups.


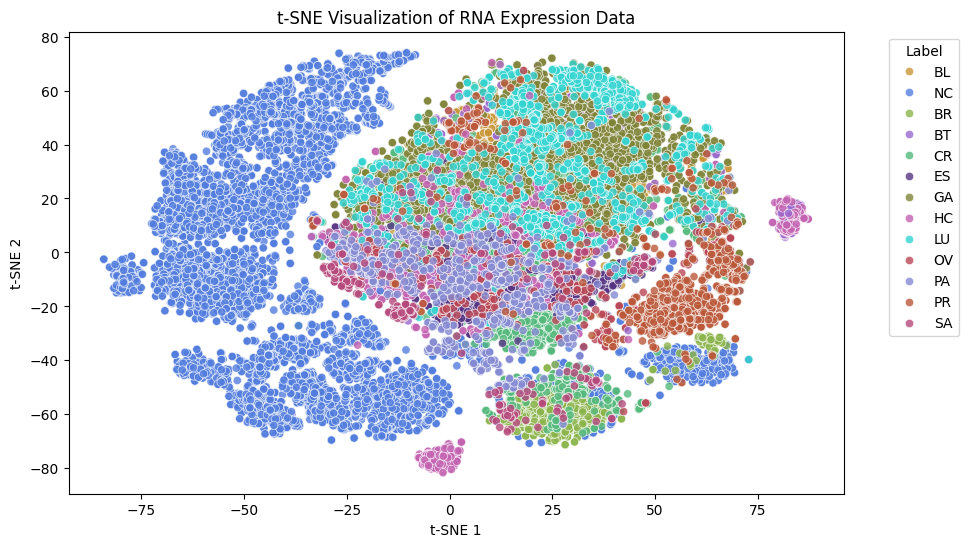


**Supplementary Figure 3.** t-distributed Stochastic Neighbor Embedding (t-SNE) plot of RNA expression data based on the 88 CFS-selected features. Each point represents a sample, colored by its corresponding label. The plot reveals nonlinear separability and local clustering among cancer types and control (NC) samples.


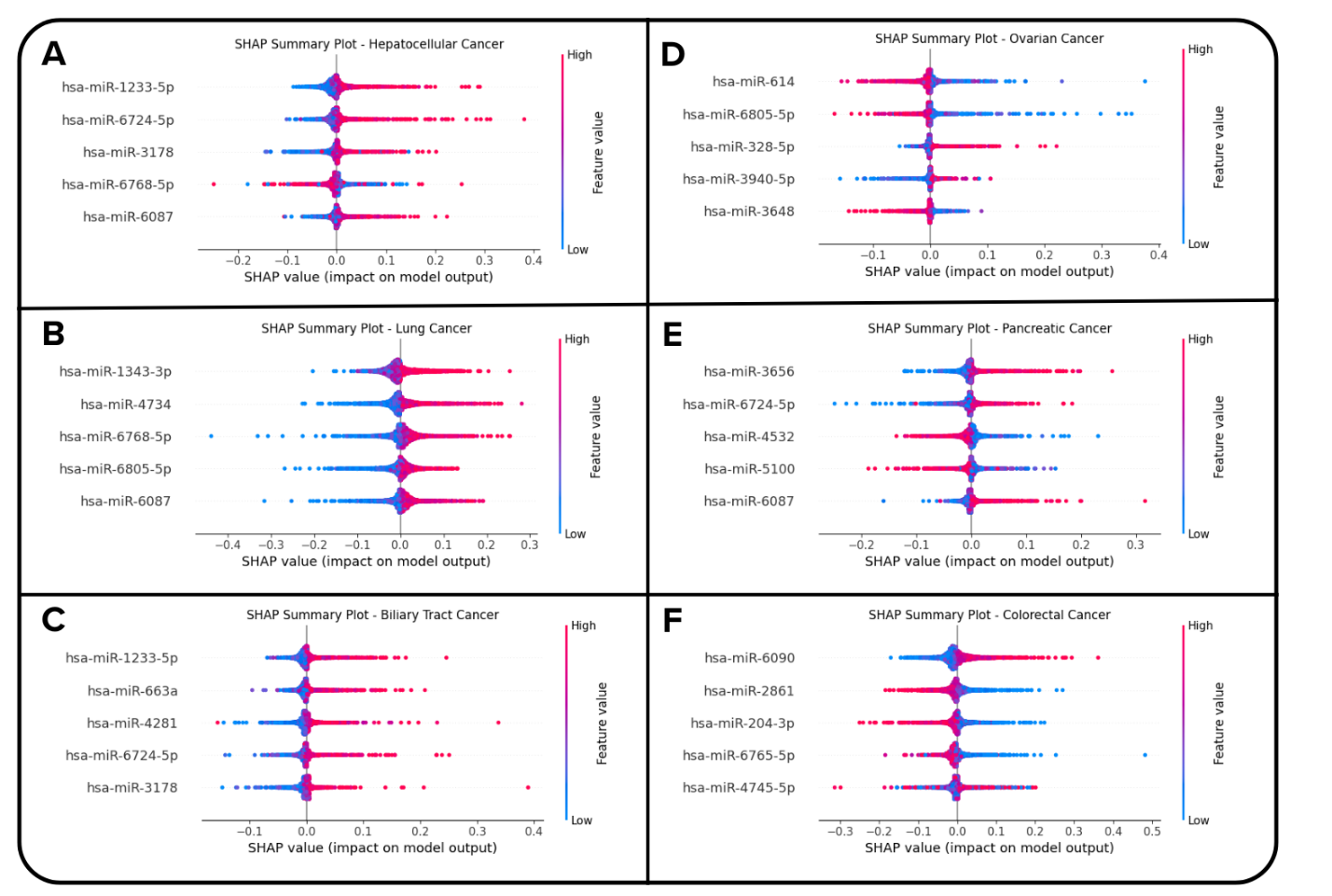


**Supplementary Figure 4.** SHAP summary plots illustrating the impact of top miRNA features on cancer-type classification models. (A–F) Each panel shows the SHAP values for the top five miRNAs in hepatocellular (A), lung (B), biliary tract (C), ovarian (D), pancreatic (E), and colorectal (F) cancers. Dots represent individual SHAP values for each sample; color indicates the feature (miRNA) expression value (red = high, blue = low). Features are ranked by their average absolute impact on model output.


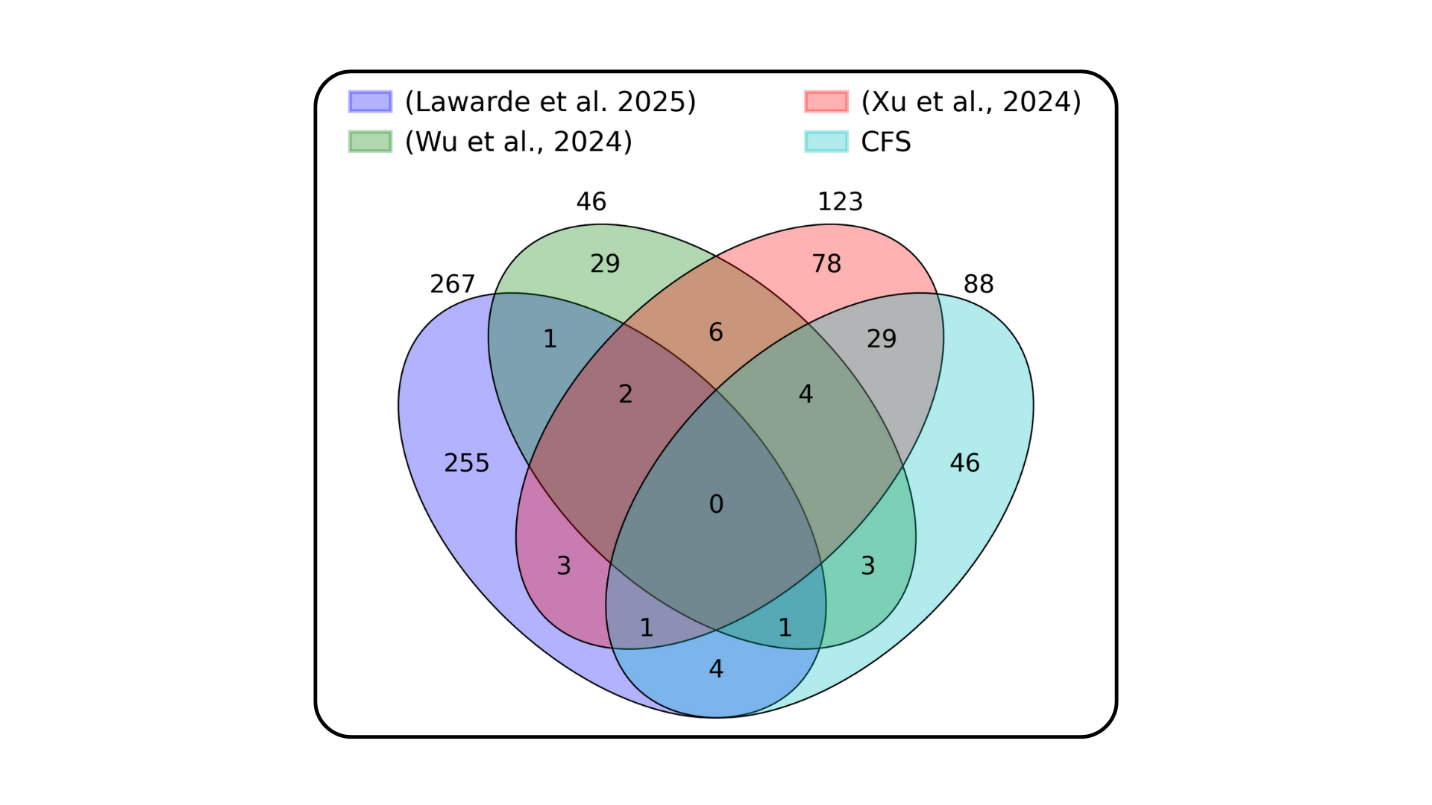


**Supplementary Figure 5.** Venn diagram comparing selected miRNA features across studies. Overlap of miRNA features identified in three previous studies, Lawarde et al. (2025), Wu et al. (2024), Xu et al. (2024), and the present study using Consensus Feature Set (CFS). The diagram highlights shared and unique miRNAs among the four sets.


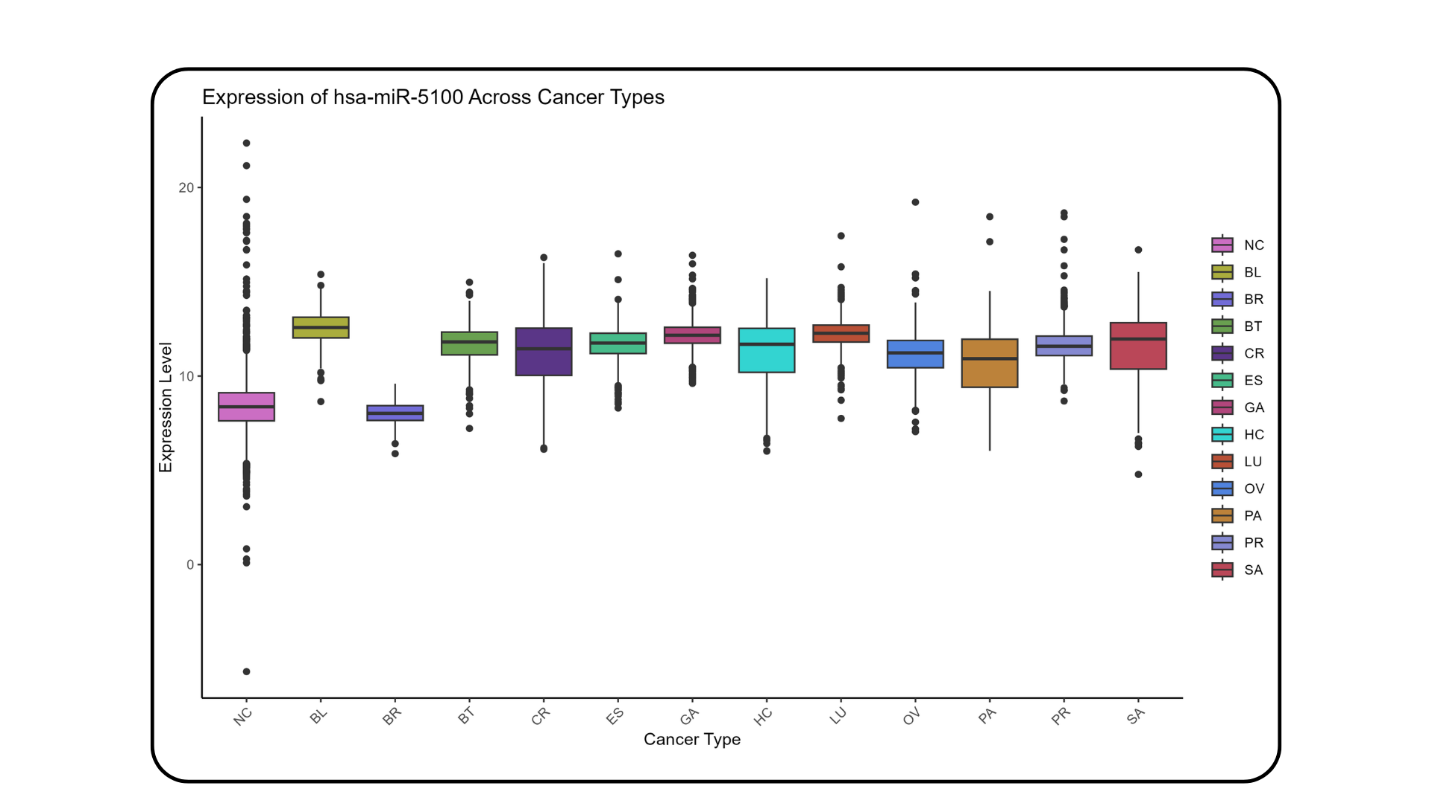


**Supplementary Figure 6.** Expression levels of hsa-miR-5100 across different cancer types. Box plot showing the distribution of hsa-miR-5100 expression in normal controls (NC) and various cancer types. Each box represents the interquartile range with the median marked, and outliers shown as individual points. Differences in expression levels highlight potential cancer-specific dysregulation of hsa-miR-5100.

1. **Supplementary Tables**

Supplementary Table-1: The important features selected using RFE with different estimators.

Supplementary Table-2: The classification report for the MLP model trained on the CFS

Supplementary Table-3: List of validated gene targets from miRecords, miRTarBase, and TarBase databases extracted using MultimiR

Supplementary Table-4: The selected features of other similar studies compared to our CFS
